# Targeting prostate cancer stem-like cells by an immunotherapeutic platform based on immunogenic peptide-sensitized dendritic cells-cytokine-induced killer cells

**DOI:** 10.1186/s13287-020-01634-6

**Published:** 2020-03-17

**Authors:** Zhu Wang, Youjia Li, Yuliang Wang, Dinglan Wu, Alaster Hang Yung Lau, Pan Zhao, Chang Zou, Yong Dai, Franky Leung Chan

**Affiliations:** 1 Department of Urology, People’s Hospital of Longhua, Southern Medical University, Shenzhen, 518109 Guangdong China; 2 School of Biomedical Sciences, Faculty of Medicine, Chinese University of Hong Kong, Shatin, Hong Kong, China; 3 Clinical Medical Research Center, The Second Clinical Medical School of Jinan University, Shenzhen People’s Hospital, Shenzhen, 518020 Guangdong China

**Keywords:** Prostate cancer stem-like cells, Dendritic cells, Cytokine-induced killer cells, Cellular immunotherapy, Cell membrane antigen

## Abstract

**Background:**

Autologous cellular immunotherapy or immune enhancement therapy has demonstrated some promising benefits for prostate cancer. T cell-based immunotherapy or sipuleucel-T therapy has yielded certain beneficial responses and a slight improvement on the overall survival of patients with metastatic castration-resistant prostate cancer (mCRPC) as shown in some clinical trials, suggesting that prostate cancer is immunoresponsive.

**Methods:**

In this study, we developed an adaptive cytokine-induced killer cell (CIK)-based immunotherapeutic application targeting the prostate cancer stem-like cells (PCSCs). In this therapeutic platform, dendritic cells (DC) were isolated from the peripheral blood mononuclear cells (PBMCs) and preloaded or sensitized with immunogenic peptides derived from two PCSC-associated cell membrane molecules, CD44 and EpCAM, followed by co-culture with the expanded peripheral blood lymphocyte (PBL)-derived CIK cells. The in vitro cytotoxic activity of DC-activated CIK cells against PCSCs was determined by CCK8 and TUNEL assays, and the in vivo anti-tumor effect of DC-activated CIK cells on prostate cancer xenograft tumors was evaluated in subcutaneous and orthotopic xenograft models.

**Results:**

Our results showed that the peptide-sensitized DC-CIK cell preparation manifested significant in vitro cytotoxic activity against the PCSC-enriched prostatospheroids and also in vivo anti-tumor effect against prostate cancer xenografts derived from the PCSC-enriched prostatospheroids.

**Conclusions:**

Together, our established immunogenic peptide-sensitized DC-CIK-based cell preparation platform manifests its potential immunotherapeutic application in targeting the PCSCs and also prostate cancer.

## Background

Prostatectomy and radiotherapy are still the mainstay treatment options for localized prostate cancer. However, a significant number of patients will develop disease recurrence and require hormone or androgen-deprivation therapy (ADT) targeting the androgen receptor (AR) signaling in androgen-sensitive prostate cancer. Although ADT is initially efficacious, almost all patients will inevitably develop resistance and metastasis as metastatic castration-resistant prostate cancer (mCRPC) shortly and with a general survival of 2–3 years. Based on this, immunotherapy has been proposed as a novel therapeutic strategy for treating advanced mCRPC. Different immunotherapeutic approaches for prostate cancer are being investigated and evaluated in clinical trials, including active immunotherapy by tumor antigen-mediated activation of antigen-presenting dendritic cells (DC), passive immunotherapy by antibodies against specific receptors or tumor antigens (e.g., prostate-specific membrane antigen/PMSA), adoptive chimeric antigen receptor T cell therapy (CART) targeting prostate cell surface antigens, adenovirus-based vaccination targeting of prostate cancer antigens (e.g., PSA and PSCA), and restoration of T cell-mediated anti-tumor activity by antibody-based blockade of immune checkpoint inhibitors (e.g., PD-1/PD-L1, CTLA-4) [1–4]. So far, sipuleucel-T (PROVENGE), based on the infusions of CD54^+^-dendritic cells activated by a recombinant prostatic acid phosphatase-granulocyte-macrophage colony-stimulating factor (PAP-GM-CSF) into patients, is the only FDA-approved immunotherapy for advanced mCRPC [5–7].

Numerous studies demonstrate and confirm the existence of a small subpopulation of cells within cancers, designated as cancer stem cells (CSCs or also called cancer stem-like cells, tumor-initiating cells, or cancer progenitor cells), being featured by their self-renewal capacity and stem cell-like characteristics. Studies in prostate cancer show that prostate cancer stem-like cells (PCSCs) are isolated from various experimental and clinical sources of prostate cancer by various methods, including low-adhesion or suspension 3D-cultures based on their anchorage-independent growth feature and antibody-based fluorescence-activated cell sorting (FACS) based on their unique expression of cell surface biomarkers (e.g., CD44, CD133, integrin α_2_β_1_) [8, 9]. Experimental studies implicate that these PCSCs, characterized by their AR^−/low^ or PSA^−/low^ features, contribute to the initiation of prostate cancer and its advanced progression to castration-resistance or resistance to ADT leading to tumor recurrence and metastasis [10, 11]. With this view, it is believed that PCSCs might be the root or origin of prostate cancer and thus curative therapy targeting PCSCs might help to eradicate this cancer.

Indeed, different experimental therapeutic strategies or approaches targeting the PCSCs have been explored in past decades. These studies also demonstrate certain beneficial effects in some preclinical models. These approaches include pharmacological inhibition of key signaling pathways associated with PCSCs (e.g., hedgehog, Wnt/β-catenin, Notch, and NF-κB) [12], manipulation of PCSC-associated miRNAs [13, 14], and also stem cell-based gene therapy [15, 16]. However, the application of immune cells or antibody-based therapy targeting the PCSCs has not been explored so far.

In this study, we established an experimental therapeutic platform of cellular immunotherapy targeting for PCSCs based on the cytokine-induced killer T cells specifically activated by dendritic cells (DC-CIK) which had been preloaded or sensitized with immunogenic peptides derived from two PCSC-associated membrane antigens, CD44 and epithelial cell adhesion molecule (EpCAM or CD326), and both have been utilized as potential therapeutic targets via different approaches for prostate cancer. Our results showed that the DC-CIK cell preparation exhibited significant in vitro cytotoxicity effect against the PCSCs and also exerted a potent in vivo anti-tumor effect in PCSC-derived xenograft models. Our results support the potential therapeutic application of this CIK-based therapeutic approach for prostate cancer immunotherapy.

## Methods

### Reagents

Recombinant human (rh) cytokines, including IL-1α, IL-2, IL-4, GM-CSF, IFN-γ, and TNF-α, and anti-CD3 and anti-CD28 antibodies were purchased from Beijing T&L Biotechnology. Fluorophore-labeled primary antibodies, including anti-CD3-FITC, anti-CD4-phycoerythrin/PE, anti-CD56-allophycocyanin/APC, anti-CD80-PE, anti-CD83-APC, and anti-CD86-PerCP-Cy5.5, were acquired from BD Biosciences; anti-CD44 and anti-EpCAM antibodies were obtained from Abcam. Ficoll-Paque PLUS medium was obtained from GE Healthcare Life Sciences; Lonza X-VIVO™ 15 medium from Fisher Scientific; CCK-8 reagent from Dojindo Molecular Technologies; and TRIzol reagent from Molecular Research Center.

### Synthetic peptides

Synthetic peptides related to human CD44 and EpCAM were designed based on their known amino acid sequences (UniProt P16070, P16422) and predicted antigenic epitope properties (including antigen index, extracellular domain, surface probability, and hydrophilicity). Three respective CD44- and EpCAM-derived peptides were synthesized (Convenience Biology, Changzhou, China) as prostate cancer stem-like cell (PCSC)-specific peptide antigens for the activation of isolated monocytes. The amino acid sequences of CD44- and EpCAM-derived synthetic peptides are listed in Supplementary Table S1.

### Cell lines and non-adherent 3D culture

Four human prostate cancer cell lines (LNCaP, 22Rv1, VCaP, and DU145) and one immortalized prostatic epithelial cell line (BPH-1) were used in this study. LNCaP, 22Rv1, and DU145 were obtained from ATCC; VCaP was provided by Dr. K. Pienta and BPH-1 from Dr. S. Hayward. The conditions of the conventional adherent 2D cultures of these cell lines were described previously [17]. Prostatospheroids enriched of PCSCs were grown and acquired using an agar-based non-adherent 3D-culture method as described previously [8]. For live-cell tracking in co-cultures, prostatic cells were also infected with an empty bicistronic lentiviral vector pWPI, which carries an IRES-EGFP cassette expressing the EGFP, followed by sorting of EGFP-positive cells by flow cytometry [17]. DU145 and 22Rv1 cells were also labeled with firefly luciferase by a lentiviral vector pLenti-Luc for orthotopic inoculation in the dorsal prostate of SCID mice and the prostate tumor xenografts formed were monitored by bioluminescence in vivo imaging (Bruker In Vivo Xtreme) [18].

### Isolation and preparation of DC and CIK cells

Peripheral blood mononuclear cells (PBMCs) or lymphocytes (PBLs) were isolated from donated blood samples of normal healthy subjects (Hong Kong Red Cross) by Ficoll-Paque PLUS density gradient centrifugation at 400*g* for 10 min, followed by culture in serum-free hematopoietic cell medium (Lonza X-VIVO™ 15 medium). After 2 h incubation, the adherent PBMCs (monocytes) were collected for dendritic cell (DC) culture and the suspended PBLs were collected for cytokine-induced killer cell (CIK) culture. The adherent monocytes were first cultured in X-VIVO 15 medium supplemented with recombinant human interleukin-4 (IL-4, 10^3^ IU/ml) for 24 h, followed by stepwise addition of granulocyte-macrophage colony-stimulating factor (GM-CSF, 10^3^ IU/ml) on day 3, TNF-α (10 ng/ml) on day 5, and finally peptide antigens (CD44- and EpCAM-derived synthetic peptides) or without on day 7 to the culture medium. CIK cells were generated from suspended PBLs following a previously described protocol with modification [19]. Briefly, the suspended PBLs were cultured in serum-free X-VIVO™ 15 medium with IFN-γ (2 × 10^3^ IU/ml), rhIL-1α (100 IU/ml), and anti-CD3 and anti-CD28 antibodies (100 ng/ml) for 7 days. After 24 h culture, rhIL-2 (10^3^ IU/ml) was added to the medium for further expansion of CIK cells. For DC-CIK cell preparation, mature DC cells (with or without peptide antigen loading) and CIK cells were mixed and co-cultured at 37 °C in a humidified atmosphere of 5% CO_2_, with one-half of the medium renewed with fresh medium supplemented with IL-2 in every 2–3 days until the CIK cells reached maturity on day 14 for harvest. For live-cell tracking in co-cultures, isolated CIK cells were labeled with CellTrace™ Far Red following the supplier’s procedure (Thermo Fisher Scientific).

### Flow cytometry analysis

Mature DC cells (with or without loading with peptide antigens) were suspended in 50 μl PBS and incubated with 5 μl of each of anti-CD80-PE, anti-CD83-APC, and anti-CD86-PerCP-Cy5.5 for 20 min at room temperature. Harvested CIK cells (upon co-culture with peptide-loaded or unloaded DC cells) were suspended in 50 μl PBS and incubated with 5 μl of each of anti-CD3-FITC, anti-CD4-PE, and anti-CD56-APC for 20 min at room temperature. After antibody incubations, the respective harvested DC and CIK cells were washed twice with PBS and re-suspended in 3 ml PBS. The cell populations were analyzed by flow cytometry (BD FACSAria II Cell Analyzer).

### Quantitative PCR and immunoblot analyses

#### Quantitative real-time RT-qPCR analysis

Total RNA was extracted from either 2D-cultured cells or 3D-cultured prostatospheroids using TRIzol reagent according to the manufacturer’s instruction, followed by reverse transcription using PrimeScript reverse transcriptase (TaKaRa Bio Inc.). Real-time PCR was performed using a SYBR green fluorescence-based method (SYBR Premix Ex Taq; TaKaRa Bio) as described previously in a real-time PCR system (StepOne, Applied Biosystems) [20]. The nucleotide sequences of primers used are listed in Supplementary Table S2.

#### Immunoblot analysis

Total cellular proteins were extracted from subconfluent cultured cells or isolated prostatospheroids using a cold lysis buffer (20 mM PIPES, 0.1% SDS, 1 mM EDTA, 1 mM EGTA, 10 mM monothioglycerol, 1 mM PMSF, 5 mM leupeptin, 0.25 M sucrose). After SDS-PAGE separation and transblotting onto PVDF membranes, resolved proteins were probed with optimally diluted primary and secondary antibodies followed by a chemiluminescence detection method (ECL Western Blotting Detection System, Amersham). The primary antibodies used are as follows: CD44 (1 M7.8.1, Abcam), EpCAM (ab71916, Abcam), and β-actin (#4970, Cell Signaling Technology).

### Cytotoxicity assay

The EGFP-labeled prostatospheroids were suspended into single cells, seeded onto 96-well plates (10^3^ cells/ml) and co-cultured with the CellTrace™ Far Red-labeled CIK cells (harvested after co-culture with peptide-loaded or unloaded DC cells) at ratios of 1:5 or 1:10 for 4 h. After co-cultures, viable cells were determined by the colorimetric cell counting kit-8 (CCK-8) assay following the manufacturer’s procedure (Dojindo Molecular Technologies, Inc.). Briefly, CCK-8 reagent or WST-8 [2-(2-methoxy-4-nitrophenyl)-3-(4-nitrophenyl)-5-(2,4-disulfophenyl)-2H-tetrazolium, monosodium salt] was added to cultured cells (10 μl, 1:10 volume) followed by 2 h incubation at 37 °C. *A*_450_ absorbance of formed WST-8 formazan was measured using a microplate spectrophotometer. The specific cytotoxicity or killing efficiency against prostate cancer cells was determined by the formula (%) = 100 × [*A*_450_ (without CIKs) − *A*_450_ (with CIKs)]/*A*_450_ (without CIKs).

### In situ TUNEL assay

Co-cultures of prostate cancer cells (derived from prostatospheroids or parental cell lines) with CIK cells (harvested after co-culture with peptide-loaded or unloaded DC cells) were seeded onto glass cover-slides and prepared as described above. After co-cultures for 1 h, apoptotic cells were detected by TUNEL labeling using a commercial kit following the manufacturer’s procedures (In situ Cell Death Detection TMR red, Sigma-Aldrich). Briefly, adherent cells were fixed with 4% paraformaldehyde in PBS, permeabilized with 0.1% Triton X-100 in 0.1% sodium citrate, and followed by incubation with the TUNEL reaction mixture containing terminal deoxynucleotidyl transferase (TdT) and TMR red-labeled dUTP. Histochemical TUNEL staining was also performed on paraffin sections of formaldehyde-fixed DU145 or 22Rv1-derived xenograft tumors, which had been injected intratumorally with either peptide-stimulated P-DC-CIK or non-stimulated NP-DC-CIK cells. TMR red-labeled apoptotic cells were detected by fluorescence microscopy with an excitation wavelength of 520–560 nm.

### In vivo tumorigenicity assay

#### Subcutaneous xenograft models

Single-cell suspensions of prostatospheroid-derived prostate cancer cells were subcutaneously injected into the flank regions of intact male SCID mice to generate xenograft tumors as described previously [18, 21]. When tumors grew to sizes of 1.0–1.5 cm^3^, host animals were randomly separated into 2 groups and treated by intratumoral injections of CIK cells (cell numbers injected 1 × 10^7^ cells/site, suspended in PBS) that had been co-cultured with peptide-loaded or unloaded DC cells. After CIK cell injections, tumor sizes were measured weekly using the formula 0.5 × width × length × height and plotted against time.

#### Orthotopic xenograft model

Luciferase-labeled DU145 cells (5 × 10^5^) were inoculated into the dorsal prostate of intact male SCID mice and allowed to grow orthotopically for 6 weeks. CIK cells were injected weekly via tail veins at the 2nd to 5th week. Orthotopic tumor growth was monitored weekly by bioluminescence in vivo imaging as described previously [18]. Data were obtained from at least three independent animals. All animal protocols were approved by the CUHK Animal Experimental Ethics Committee and performed in accordance with the guidelines.

### Statistical analysis

Data were expressed as mean ± SD. Differences of results were evaluated with two-tailed Student’s *t* test and considered significant where *P* values < 0.05.

## Results

### 3D-cultured prostatospheroids exhibit increased expressions of CD44 and EpCAM

**Fig. 1:**
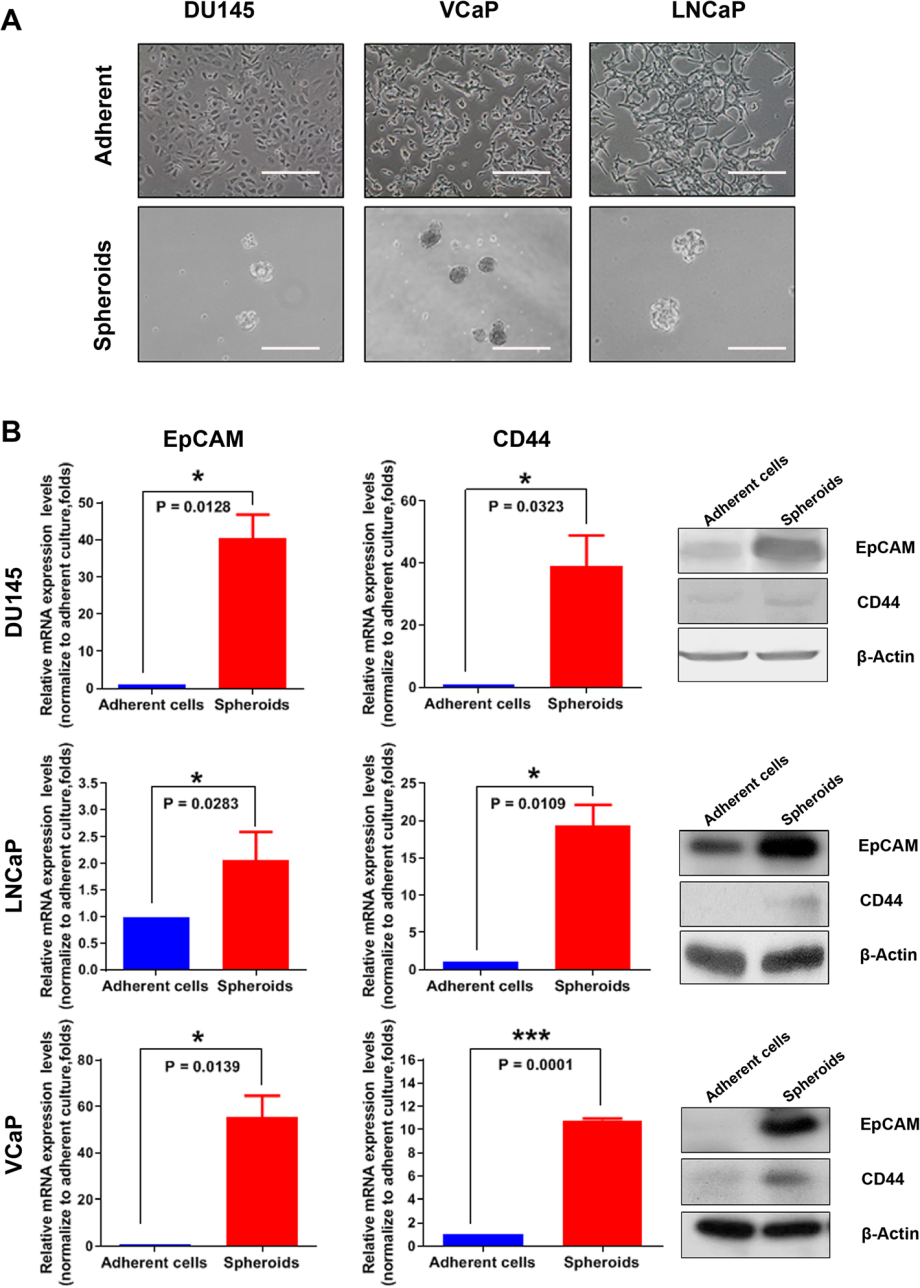
Non-adherent 3D-cultured prostatospheroids exhibit increased expressions of two PCSC-associated surface markers EpCAM and CD44. **a** Representative images of three selected prostate cancer cell lines (AR-positive, VCaP and LNCaP; AR-negative, DU145) grown under the adherent 2D-culture condition (magnification, × 20; bars, 100 μm) and the non-adherent 3D-culture on agar-coated surfaces (magnification, × 20; bars, 200 μm). **b** RT-qPCR and immunoblot analyses of EpCAM and CD44. Results showed that all prostatospheroids derived from different prostate cancer cells expressed significantly higher mRNA and protein levels of EpCAM and CD44 as compared to their counterpart adherent cultured cells. *P < 0.05 versus adherent cultured cells

Based on the common self-renewal and anchorage-independent growth capacities of CSCs derived from different cancers, we have previously established an agar-based non-adherent 3D-culture method for isolation and enrichment of CSCs [8]. Under this 3D-culture condition, the prostatospheroids derived from various prostate cancer cell lines and primary prostate cancer tissues are enriched of PCSCs. Here, we analyzed the expression patterns of two PCSC-associated membrane markers, CD44 and EpCAM [22, 23], in 3D-cultured prostatospheroids derived from three different prostate cancer cell lines (AR-positive LNCaP and VCaP; AR-negative DU145). Our results showed that the PCSC-enriched prostatospheroids expressed significantly higher mRNA and protein levels of both CD44 and EpCAM as compared to their counterpart cells grown under the conventional adherent 2D-culture condition (Fig. 1). Based on their increased expression patterns, we decided to select CD44 and EpCAM as the target antigens for the synthesis of immunogenic peptides and their use for stimulation of DC cells.

### Generation of peptide antigen-sensitized DC-CIK cell preparation

**Fig. 2:**
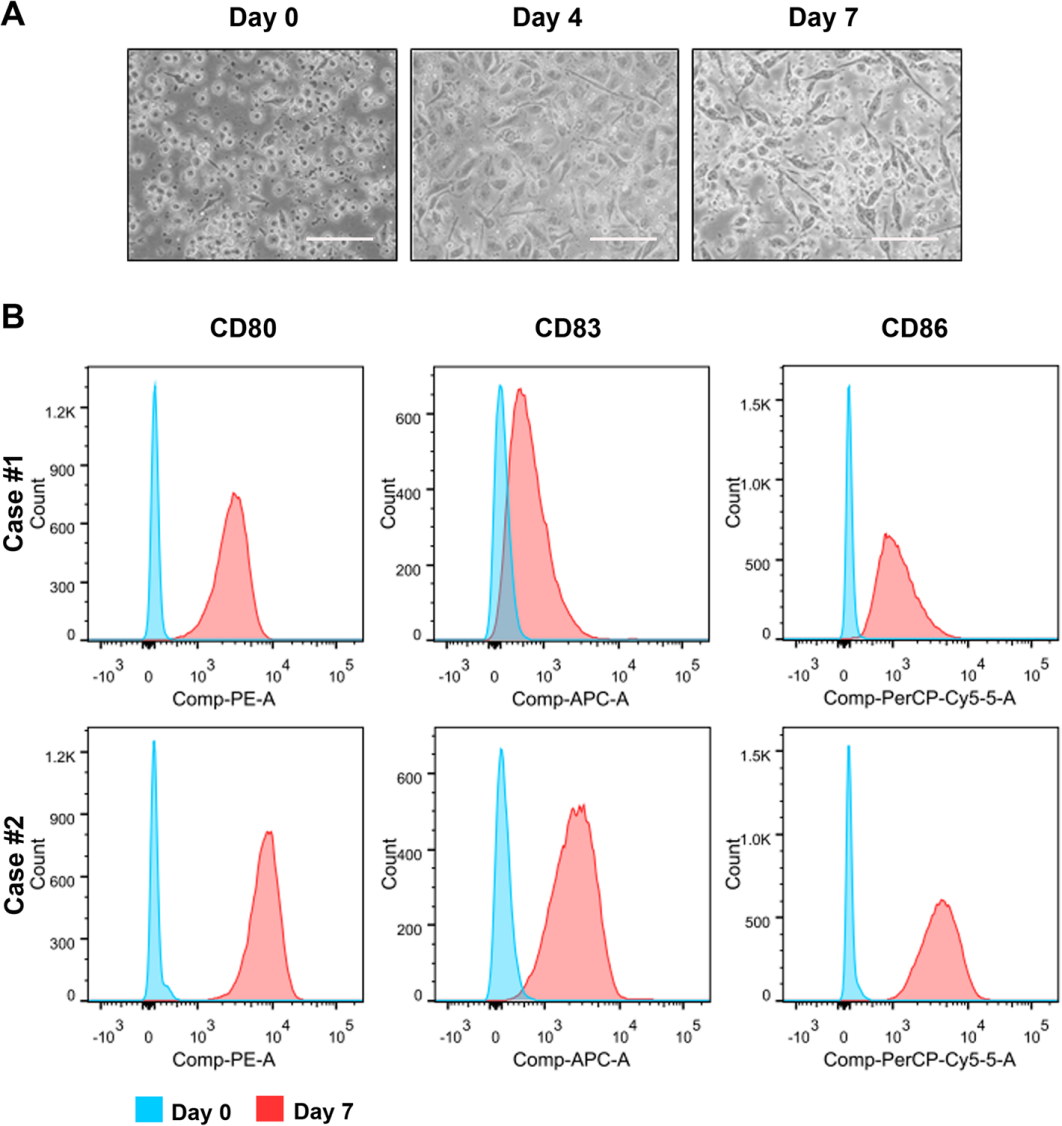
Phenotypic characterization of DC cells. **a** Representative images of adherent cultured PBMC-isolated precursor monocytes (day 0), inflammatory cytokine-stimulated maturing DC cells (day 4), and peptide-loaded mature DC cells (day 7). The peptide-sensitized DC cells exhibited the typical dendritic appearance. Magnification, × 40; bars, 200 μm. **b** FACS analysis. Results showed that the peptide-stimulated DC cells, isolated from two representative PBMC preparations, expressed higher levels of three DC-associated surface markers, CD80, CD83, and CD86

**Fig. 3:**
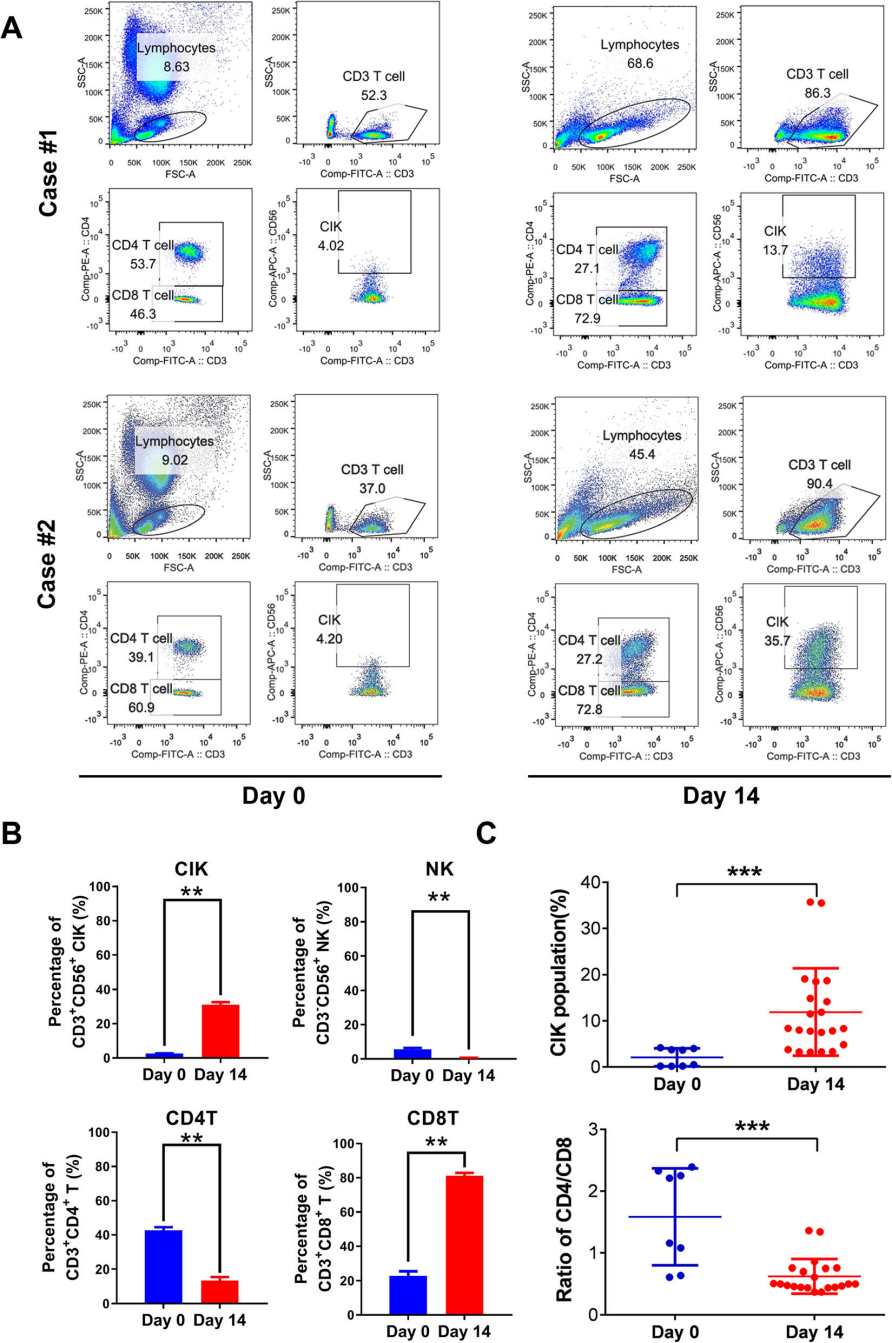
FACS analysis of DC-activated CIK cells. **a** FACS analysis of CD3^+^CD56^+^ CIK subset cells upon co-culture of CIK cells with peptide-sensitized DC cells (on day 0 and day 14) in two representative preparations from PBLs. Results showed that the population of CD3^+^CD56^+^ CIK cells showed a significant increase after a 14-day DC-CIK co-culture. **b** FACS analysis showed that the CIK cells on day 14 co-culture contained less CD3^+^CD4^+^ T cells (CD4 T cells) and CD3^−^CD56^+^ NK cells but more CD3^+^CD4^−^ T cells (CD8 T cells) as compared to CIK cells on day 0 culture. **c** FACS analysis of CIK cell population from a total of 21 samples of PBLs. Summary results showed that the modified CIK culture protocol with stimulation by peptide-loaded DC cells could generate more population of CIK cells and decreased ratio of CD4^+^/CD8^+^ T cells. **P < 0.01; ***P < 0.001 versus cells collected on day 0

Maturing DC cells were prepared from the adherent cultured PBMCs upon stimulation by inflammatory cytokines. On day 7 culture, DC cells were further sensitized by exposure to CD44- and EpCAM-derived synthetic peptides and the peptide-loaded DC cells displayed the characteristic dendritic morphology (Fig. 2a). FACS analysis validated that the DC cells expressed significantly higher levels of mature DC-specific surface markers (including CD80, CD83, and CD86) as compared to their precursor monocytes (Fig. 2b). CIK cells were prepared from the suspended PBLs following stimulation with cytokines (IFN-γ, IL-1α, and IL-2) and CD3 plus CD8 antibodies for their induction of cytotoxic activity and expansion. CIK cells were further co-cultured or activated with the peptide-loaded or unloaded DC cells for another 7 days as DC-activated or DC-stimulated CIK cell preparation. Most mature DC cells died at day 7 co-culture when the CIK cells were harvested. The PBL-derived lymphocytes without cytokine stimulation (day 0 culture) and the CIK cells with DC induction were analyzed by FACS on their cytotoxic T lymphocyte (CTL) subpopulations and cytotoxicity (Figs. 3a, b). Results showed that the DC-stimulated CIK cells contained more population of CD3^+^CD56^+^ subset (14–36%), which are characterized to display higher cell proliferation rates and more potent cytotoxicity against tumor cells [24], as compared to PBL-derived lymphocytes without cytokine stimulation (4.02%). FACS analysis also showed that the DC-stimulated CIK cells contained less subsets of CD3^+^CD4^+^ T cells (CD4 T cells) and CD3^−^CD56^+^ NK cells but more population of CD3^+^CD4^−^ T cells (CD8 cytotoxic T cells), as compared to PBL-derived lymphocytes without cytokine and DC stimulation (Fig. 3b). The DC-stimulated CIK cells prepared from all PBL samples (totally *n* = 21 blood samples) by this protocol consistently contained increased population of CD3^+^CD56^+^ subset and decreased ratio of CD4 to CD8 T cell subsets (Fig. 3c).

### In vitro cytotoxic activity of DC-activated CIK cells against PCSCs

**Fig. 4:**
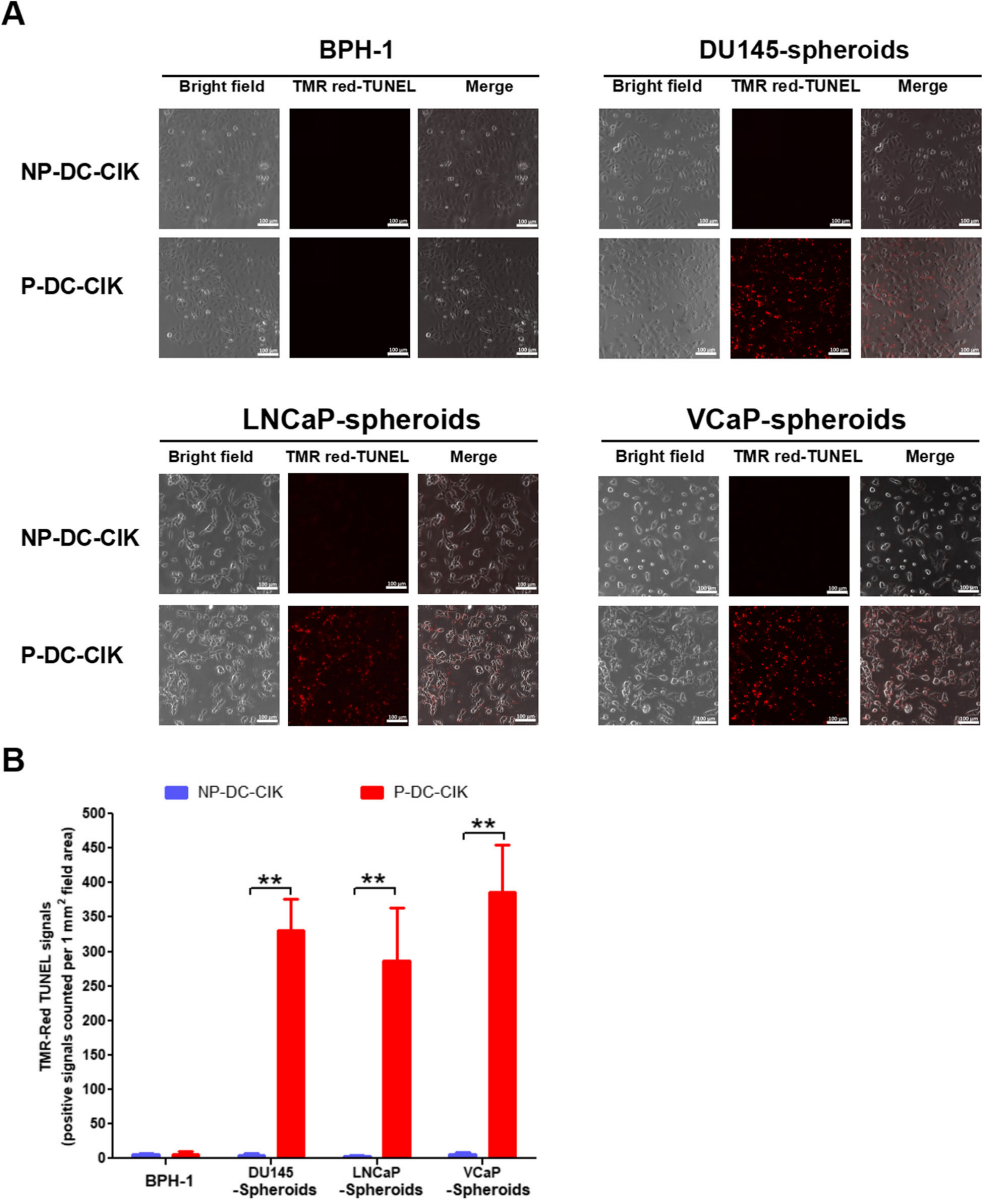
Detection of apoptotic cells in prostatic cells upon 1 h incubation with P-DC-CIK or NP-DC-CIK by TMR red-based TUNEL assay. **a** Representative images show the TUNEL-positive apoptotic cells induced in prostatospheroid-derived prostate cancer cells. **b** Graph shows the quantitation of TMR red signals detected in prostatic cells upon co-cultures with P-DC-CIK or NP-DC-CIK cells. Results showed that significant increases of TMR red-labeled apoptotic cells were detected in prostatospheroid-derived prostate cancer cells (DU145, LNCaP, and VCaP) upon co-culture with the P-DC-CIK as compared to the NP-DC-CIK control. Results also showed that no TMR red-labeled apoptotic cells were detected among BPH-1 immortalized non-malignant prostatic epithelial cells upon incubation with either P-DC-CIK or NP-DC-CIK. Magnification, × 20; bars, 100 μm. *NP-DC-CIK* non peptide-sensitized dendritic cells-cytokine-induced killer cell preparation, *P-DC-CIK* peptide-sensitized dendritic cells-cytokine-induced killer cell preparation

**Fig. 5:**
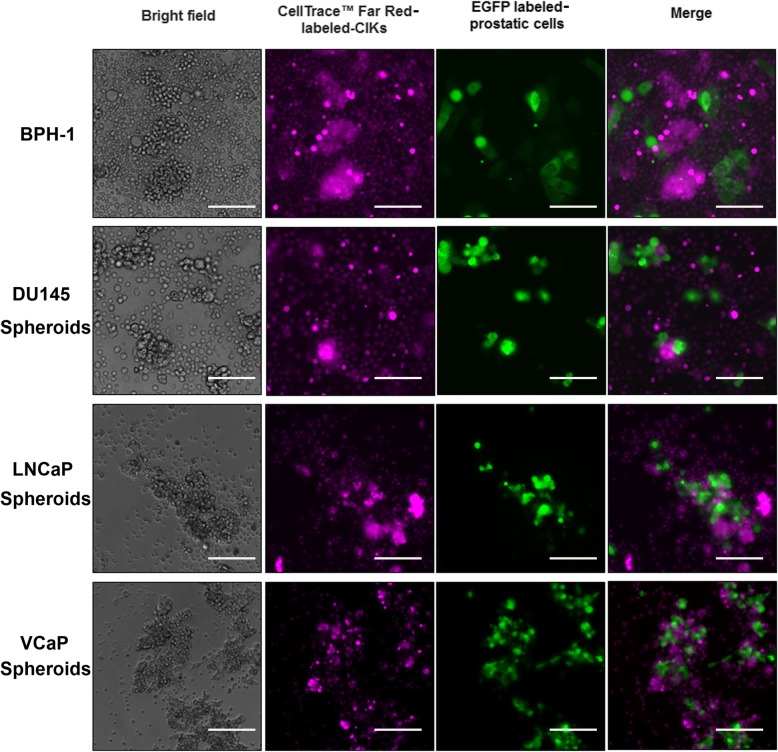
Microscopic evaluation of the cytotoxic activity of CIK cells prepared from co-culture with peptide-loaded DC cells against the BPH-1 immortalized prostatic epithelial cells or prostatospheroid-derived prostate cancer cells. Representative fluorescence images show the co-cultures of CellTrace™ Far Red-labeled peptide-loaded DC-activated CIK cells and the EGFP-labeled BPH-1 cells or prostate cancer cells derived from DU145, LNCaP, or VCaP prostatospheroids. Cytochemical examination revealed that a significant induction of apoptotic cells, as evidenced by their fragmented condensed nuclei, was demonstrated among the prostatospheroid-derived prostate cancer cells upon 4 h incubation with the peptide-loaded DC-activated CIK cells. However, no obvious apoptotic cells were detected among the BPH-1 cells after co-culture with the same P-DC-CIK cell preparation

**Fig. 6:**
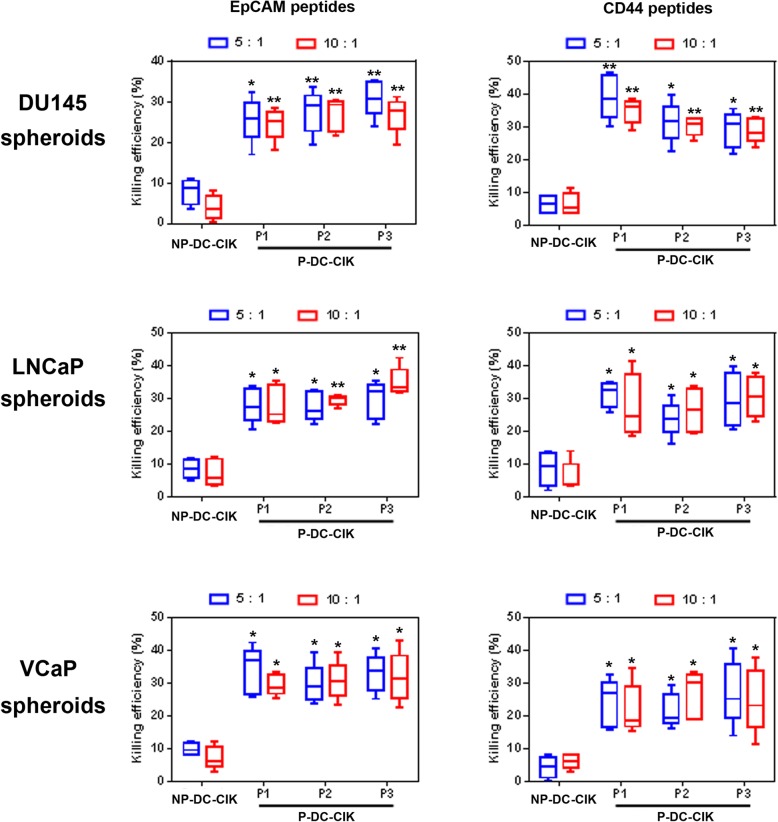
Cytotoxicity or cancer cell killing efficiency of peptide-loaded DC-activated CIK cells against the prostatospheroid-derived prostate cancer cells as determined by CCK-8 cell viability assay followed by *A*_450_ measurement. Results showed that the peptide-loaded P-DC-CIK cells exhibited significantly higher cancer cell killing efficiency against the prostate cancer cells derived from DU145, LNCaP, or VCaP prostatospheroids than the peptide-unloaded NP-DC-CIK cells (control). No significant difference on the cytotoxicity against the prostate cancer cells was observed among the different EpCAM- and CD44-derived peptides (P1, P2, and P3) and also between effect-target ratios of 5:1 and 10:1. *P < 0.05; **P < 0.01 versus peptide-unloaded NP-DC-CIK cells

PCSCs were isolated from the non-adherent 3D-cultured prostatospheroids derived from three different prostate cancer cell lines (AR-positive, LNCaP and VCaP; AR-negative, DU145). To determine the specific cytotoxicity of peptide-loaded DC-activated CIK cells against the PCSCs, the effector P-DC-CIK cells were co-cultured with the target cells (PCSCs or control BPH-1 immortalized prostatic epithelial cells) at an effector-target ratio of 10:1 for 1 h followed by in situ TUNEL assay for the detection of apoptotic cells. Results showed significant induction of apoptotic cells was detected in prostate cancer cells prepared from the DU145-, LNCaP-, or VCaP-derived prostatospheroids upon incubation with P-DC-CIK but not NP-DC-CIK cells (Fig. 4). However, no obvious apoptotic cells were detected among the BPH-1 cells after co-culture with the P-DC-CIK cells. Moreover, the induction of apoptotic cells were also examined in co-cultures of P-DC-CIK cells (CellTrace™ Far Red-labeled as effector cells) with prostatic cells (EGFP-labeled prostatospheroid-derived prostate cancer cells or BPH-1 immortalized prostatic epithelial cells as target cells) at an effector-target ratio of 10:1 for 4 h followed by fluorescence microscopy observation of apoptotic cells. Results revealed that significant induction of apoptotic cells with typically condensed fragmented nuclei was observed among the prostatospheroid-derived prostate cancer cells upon incubation with the peptide-loaded DC-activated CIK cells. However, no obvious apoptotic cells were detected among the BPH-1 cells after co-culture with the peptide-loaded DC-activated CIK cells (Fig. 5). Furthermore, to evaluate the cancer cell killing efficiency of peptide-loaded DC-activated CIK cells against prostatospheroids or PCSCs, the effector DC-stimulated CIK cells were co-cultured with the target PCSC-enriched prostatospheroids at an effector-target ratio of 5:1 or 10:1 for 4 h followed by the CCK-8 cell viability assay and *A*_450_ absorbance measurement. Results showed that the P-DC-CIK cells activated by the CD44- or EpCAM-derived peptides (P1–P3) could induce significant killing efficiencies against prostatospheroids as compared to non-peptide-loaded NP-DC-CIK cells, and also no significant differences on killing efficiency were seen between the co-culture conditions with effector-target ratios of 5:1 or 10:1 (Fig. 6). Together, these results indicated that the peptide-loaded DC-activated CIK cells exhibited specific cytotoxicity or killing efficiency against prostatospheroid-derived prostate cancer cells but not the immortalized prostatic epithelial cells.

### Anti-tumor effect of DC-activated CIK cells on prostate cancer xenograft tumors

**Fig. 7:**
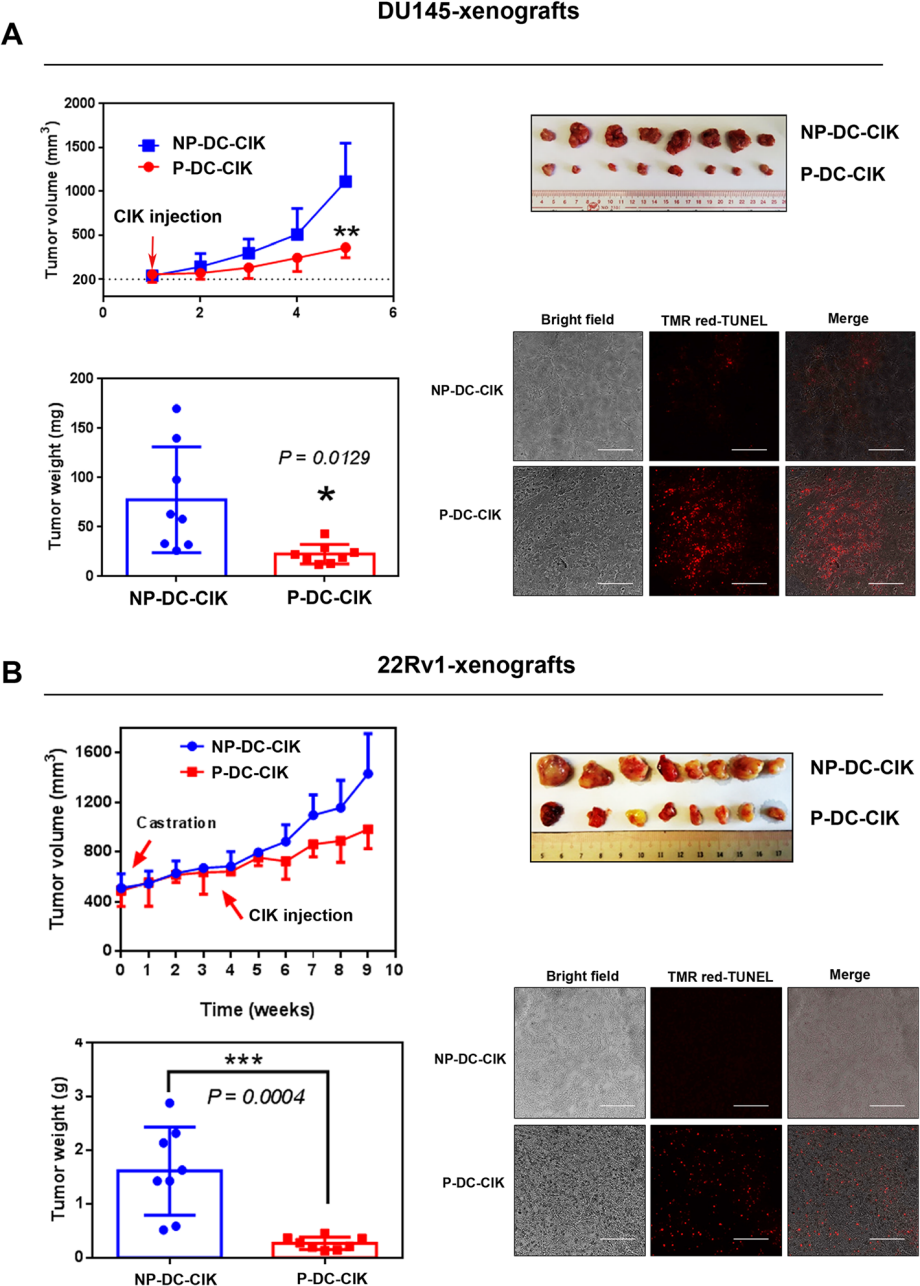
Anti-tumor activity of intratumoral injection of peptide-loaded DC-activated CIK cell preparations (P-DC-CIK) or non-peptide-loaded NP-DC-CIK cell preparations on two prostate cancer xenograft models derived from prostatospheroids. **a** DU145 xenografts. Upon intratumoral injection of P-DC-CIK, DU145 xenograft tumors grew very slowly. **b** 22Rv1 xenografts. Castration-refractory 22Rv1-CRPC xenografts were induced in SCID mice bearing 22Rv1 xenografts by castration when the tumor sizes reached about 5 mm^3^. Similar to DU145 xenografts, intratumoral injection of P-DC-CIK could significantly suppress the tumor growth as compared to injection of NP-DC-CIK control. Histochemical TUNEL staining revealed that significant increases of TMR red-labeled apoptotic cells were detected in both DU145 and 22Rv1 xenografts treated with P-DC-CIK as compared to NP-DC-CIK. *P < 0.05; ***P < 0.001 versus NP-DC-CIK control

**Fig. 8:**
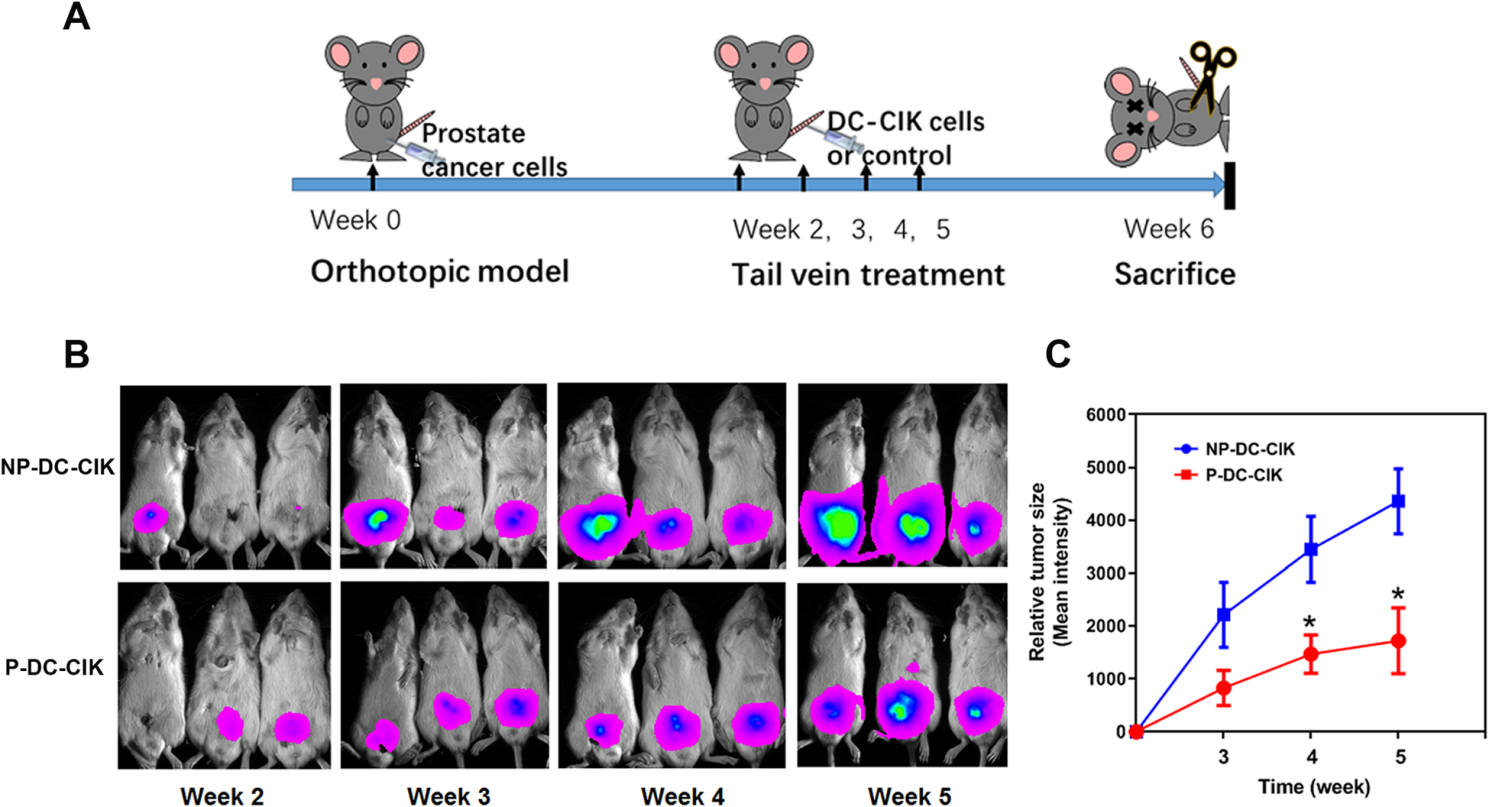
Anti-tumor activity of tail vein injection of peptide-loaded DC-activated CIK cell preparations (P-DC-CIK) on DU145-derived orthotopic xenograft tumors. **a** Schematic diagram shows the treatment scheme of tail vein injection of P-DC-CIK or NP-DC-CIK cell preparations in the DU145-derived orthotopic prostate cancer xenograft model. Luciferase-labeled DU145-Luc^+^ cells were injected into the dorsal prostate of intact male SCID mice followed by orthotopic growth for 2 weeks. P-DC-CIK or NP-DC-CIK cells were injected weekly via tail vein at the 2nd to 5th week. Orthotopic tumor growth was monitored weekly by chemiluminescence in vivo imaging. **b** Representative bioluminescent images of mice at 2nd to 5th week post-intraprostatic inoculation of DU145-Luc^+^ cells with tail vein injection of DC-CIK cell preparations. **c** Growth curve of DU145-Luc^+^ xenograft tumors. Results showed that the tail injection of P-DC-CIK but not the NP-DC-CIK control could moderately suppress the orthotopic tumor growth of DU145-Luc^+^ cells

We have previously demonstrated that the PCSC-enriched prostatospheroids exhibit higher tumorigenicity as compared with their parental prostate cancer cells prepared the conventional 2D-adherent culture [8]. We next evaluated the potential anti-tumor effect of peptide-sensitized DC-activated CIK cells on xenograft tumors formed by low cell number injections of cells prepared from DU145- or 22Rv1-derived prostatospheroids. Results showed that intratumoral injection of peptide-sensitized DC-activated CIK cells could significantly suppress the tumor growth of DU145-xenograft tumors grown in intact host mice and also the castration-relapse 22Rv1-CRPC xenograft tumors grown in castrated mice (Fig. 7). Histochemical in situ TUNEL staining also revealed that the xenograft tumors treated with intratumoral injection of peptide-loaded P-DC-CIK cells exhibited more significant TUNEL signals of apoptotic cells or DNA cleavage than that treated with the non-peptide-loaded NP-DC-CIK cells, suggesting that intratumoral injections of peptide-stimulated P-DC-CIK cells could induce apoptosis of tumor cells in prostate xenograft tumors. We also evaluated the anti-tumor effect of peptide-loaded DC-activated CIK cells via tail vein injection on an orthotopic xenograft model of DU145 cells. Results showed that tail vein injection of peptide-loaded DC-activated CIK cells could also moderately inhibit the orthotopic tumor growth of DU145 xenograft tumors but less effective as compared to treatment by intratumoral injection (Fig. 8).

## Discussion

Although immunotherapy by different targeting approaches has demonstrated certain benefits on the overall or progression-free survival to mCRPC patients in some clinical trials, the clinical responses are still inconsistent or in failure [25]. One possible reason could be due to the presence of small population of therapy-resistant PCSCs. In the present study, we establish a novel therapeutic platform or strategy of cellular immunotherapy targeting the PCSCs using a DC-CIK cell preparation that has been preloaded or sensitized by immunogenic peptides derived from two PCSC-associated membrane antigens, CD44 and EpCAM. Our results showed that the immunogenic peptide-sensitized DC-CIK cell preparation exhibited significant in vitro cytotoxicity against prostate cancer cells derived from the PCSC-enriched prostatospheroids and also anti-tumor efficacy against the prostatospheroid-derived xenograft tumors. Our results demonstrate the potential therapeutic value of CIK-based immunotherapy targeting the EpCAM^+^ and CD44^+^ prostate cancer cells or PCSCs.

EpCAM is a cell membrane glycoprotein highly expressed in primary and metastatic prostate cancer as compared to normal and benign hyperplastic prostates [26, 27] and is also considered as a CSC biomarker for multiple cancer types [28–30]. Its elevated expression is closely associated with metastasis and poor prognosis of prostate cancer [31]. For diagnosis application, EpCAM is used as a specific membrane biomarker for antibody-based isolation of circulating tumor cells from mCRPC patients [32, 33]. EpCAM has been evaluated in some preclinical models as an immunotherapeutic target in CART-based and antibody-based immunotherapy for prostate cancer. Deng et al. [34] show that the EpCAM-specific CAR-transduced PBLs exhibit cytotoxic effects against the PC-3M prostate cancer cells in vitro and in vivo. Bispecific EpCAM-CD3/CD16 antibody has been evaluated on its anti-tumor activity on PC-3 prostate cancer cells in vitro and in vivo xenografts [35, 36]. However, clinical trials based on EpCAM-CART and EpCAM-antibody immunotherapy for prostate cancer are still absent so far.

CD44 is also a cell membrane glycoprotein functionally acting as a receptor for hyaluronic acid (HA) and plays roles in cell adhesion and migration. Although CD44 and its spliced variants exhibit downregulation in high-grade and metastatic prostate cancer [37–41], their altered expression patterns show a correlation to prognosis in prostate cancer [42–45]. CD44 is characterized as a PCSC biomarker and isolated CD44^+^ prostate cancer cells from various sources show higher tumorigenicity and metastatic potentials [46–48]. CD44 has been investigated as a potential therapeutic target for prostate cancer. Application of HA-coated or CD44 antibody-coated nanoparticles or liposomes is utilized for specific delivery of chemotherapeutic drugs or bioactive compounds targeting the CD44^+^ prostate cancer cells [49–51].

In this study, we observed that the anti-tumor efficacy on prostate cancer xenograft tumors by tail vein injection of peptide-loaded DC-activated CIK cell preparation was less effective as compared to that by intratumoral injection of the same cell preparation. The prostate cancer immune microenvironment is shown to be immunosuppressive, as shown by the recruitment and accumulation of T regulatory cells (CD4^+^ Tregs) and T_H_17 lymphocytes [52, 53], myeloid-derived suppressive cells [54], NK cells (CD65^+^) with low or no cytotoxic activity, and also elevated levels of secreted TGFβ1 [55]. This preexisting immunosuppressive or hostile microenvironment in prostate tumor bed may be a potential hindrance to the infiltration of the infused sensitized DC-CIK cell preparations and thus attenuate their anti-tumor or anti-PCSC activity, and it remains to be determined and overcome. On the other hand, the immune environment in primary tumors of prostate cancer can be modulated by therapies. It is shown that ADT can enhance the accumulation of T lymphocytes (CD3^+^, CD8^+^) and CD68^+^ macrophages and thus induce some short-term beneficial immune responses [56, 57]. This also provides the rationale that the combination of immunotherapy with ADT may offer additional or synergistic efficacy in the treatment of prostate cancer. But it remains to further investigate whether the combination of the present established immunogenic peptide-sensitized DC-CIK therapeutic platform with ADT could maximize the efficacy on prostate cancer treatment.

## Conclusions

Here, we developed and optimized a novel adaptive immunotherapeutic platform targeting the PCSCs, based on the DC-CIK cell preparation that has been pre-sensitized with immunogenic peptides derived from two PCSC-associated surface antigens, EpCAM and CD44. Our results showed that the peptide-sensitized DC-CIK cell preparation could manifest a significant in vitro cytotoxic activity against the PCSC-enriched prostatospheroids and also in vivo anti-tumor effect against prostate cancer xenografts derived from the PCSC-enriched prostatospheroids or prostate cancer cells. Our present study demonstrates the potential immunotherapeutic application of the CIK cells, upon DC-sensitization by PCSC-derived immunogenic peptides, for targeting the PCSCs or advanced metastatic prostate cancer.

## Supplementary information

**Additional file 1: Tables S1 and S2.** Sequence information of CD44- and EpCAM-derived synthetic peptides and nucleotide sequence of PCR primers used.

## Data Availability

All data generated or analyzed in this study are included in this article.

## Abbreviations

3D culture: Three-dimensional culture
ADT: Androgen-deprivation therapy
AR: Androgen receptor
CART: Chimeric antigen receptor T cell therapy
CIK: Cytokine-induced killer cell
CRPC: Castration-resistant prostate cancer
DC: Dendritic cells
PCSCs: Prostate cancer stem cells
PSA: Prostate-specific antigen
PSMA: Prostate-specific membrane antigen

## References

[1] Kiessling A, Wehner R, Fussel S, Bachmann M, Wirth MP, Schmitz M (2012). Tumor-associated antigens for specific immunotherapy of prostate cancer. Cancers (Basel)..

[2] Comiskey MC, Dallos MC, Drake CG (2018). Immunotherapy in prostate cancer: teaching an old dog new tricks. Curr Oncol Rep.

[3] Lee P, Gujar S (2018). Potentiating prostate cancer immunotherapy with oncolytic viruses. Nat Rev Urol.

[4] Venturini Nicholas J., Drake Charles G. (2018). Immunotherapy for Prostate Cancer. Cold Spring Harbor Perspectives in Medicine.

[5] Cheever MA, Higano CS (2011). PROVENGE (sipuleucel-T) in prostate cancer: the first FDA-approved therapeutic cancer vaccine. Clin Cancer Res.

[6] Sims RB (2012). Development of sipuleucel-T: autologous cellular immunotherapy for the treatment of metastatic castrate resistant prostate cancer. Vaccine..

[7] Wesley JD, Whitmore J, Trager J, Sheikh N (2012). An overview of sipuleucel-T: autologous cellular immunotherapy for prostate cancer. Hum Vaccin Immunother.

[8] Gao W, Wu D, Wang Y, Wang Z, Zou C, Dai Y (2018). Development of a novel and economical agar-based non-adherent three-dimensional culture method for enrichment of cancer stem-like cells. Stem Cell Res Ther.

[9] Skvortsov S, Skvortsova II, Tang DG, Dubrovska A (2018). Concise review: prostate cancer stem cells: current understanding. Stem Cells.

[10] Ojo D, Lin X, Wong N, Gu Y, Tang D (2015). Prostate cancer stem-like cells contribute to the development of castration-resistant prostate Cancer. Cancers (Basel).

[11] Mei Wenjuan, Lin Xiaozeng, Kapoor Anil, Gu Yan, Zhao Kuncheng, Tang Damu (2019). The Contributions of Prostate Cancer Stem Cells in Prostate Cancer Initiation and Metastasis. Cancers.

[12] Leao R, Domingos C, Figueiredo A, Hamilton R, Tabori U, Castelo-Branco P (2017). Cancer stem cells in prostate cancer: implications for targeted therapy. Urol Int.

[13] Fang YX, Chang YL, Gao WQ (2015). MicroRNAs targeting prostate cancer stem cells. Exp Biol Med (Maywood).

[14] Yun EJ, Lo UG, Hsieh JT (2016). The evolving landscape of prostate cancer stem cell: therapeutic implications and future challenges. Asian J Urol.

[15] Kim JH, Lee HJ, Song YS (2014). Stem cell based gene therapy in prostate cancer. Biomed Res Int.

[16] Souza AG, Bastos VAF, Silva IBB, Marangoni K, Goulart VA (2016). Different gene therapy strategies: a overview for prostate cancer. Curr Gene Ther.

[17] Yu S, Xu Z, Zou C, Wu D, Wang Y, Yao X (2014). Ion channel TRPM8 promotes hypoxic growth of prostate cancer cells via an O2 -independent and RACK1-mediated mechanism of HIF-1alpha stabilization. J Pathol.

[18] Cai G, Wu D, Wang Z, Xu Z, Wong KB, Ng CF (2017). Collapsin response mediator protein-1 (CRMP1) acts as an invasion and metastasis suppressor of prostate cancer via its suppression of epithelial-mesenchymal transition and remodeling of actin cytoskeleton organization. Oncogene..

[19] Schmidt-Wolf IG, Negrin RS, Kiem HP, Blume KG, Weissman IL (1991). Use of a SCID mouse/human lymphoma model to evaluate cytokine-induced killer cells with potent antitumor cell activity. J Exp Med.

[20] Wang Z, Wu D, Ng CF, Teoh JY, Yu S, Wang Y (2018). Nuclear receptor profiling in prostatospheroids and castration-resistant prostate cancer. Endocr Relat Cancer.

[21] Yu S, Wang X, Ng CF, Chen S, Chan FL (2007). ERRgamma suppresses cell proliferation and tumor growth of androgen-sensitive and androgen-insensitive prostate cancer cells and its implication as a therapeutic target for prostate cancer. Cancer Res.

[22] Palapattu GS, Wu C, Silvers CR, Martin HB, Williams K, Salamone L (2009). Selective expression of CD44, a putative prostate cancer stem cell marker, in neuroendocrine tumor cells of human prostate cancer. Prostate..

[23] Imrich S, Hachmeister M, Gires O (2012). EpCAM and its potential role in tumor-initiating cells. Cell Adhes Migr.

[24] Pievani A, Borleri G, Pende D, Moretta L, Rambaldi A, Golay J (2011). Dual-functional capability of CD3+CD56+ CIK cells, a T-cell subset that acquires NK function and retains TCR-mediated specific cytotoxicity. Blood..

[25] Cordes LM, Gulley JL, Madan RA (2018). Perspectives on the clinical development of immunotherapy in prostate cancer. Asian J Androl.

[26] Massoner P, Thomm T, Mack B, Untergasser G, Martowicz A, Bobowski K (2014). EpCAM is overexpressed in local and metastatic prostate cancer, suppressed by chemotherapy and modulated by MET-associated miRNA-200c/205. Br J Cancer.

[27] Ni J, Cozzi P, Hao J, Beretov J, Chang L, Duan W (2013). Epithelial cell adhesion molecule (EpCAM) is associated with prostate cancer metastasis and chemo/radioresistance via the PI3K/Akt/mTOR signaling pathway. Int J Biochem Cell Biol.

[28] Visvader JE, Lindeman GJ (2008). Cancer stem cells in solid tumours: accumulating evidence and unresolved questions. Nat Rev Cancer.

[29] Gires O, Klein CA, Baeuerle PA (2009). On the abundance of EpCAM on cancer stem cells. Nat Rev Cancer.

[30] Munz M, Baeuerle PA, Gires O (2009). The emerging role of EpCAM in cancer and stem cell signaling. Cancer Res.

[31] Netsch C, Knipper S, Bach T, Herrmann TR, Gross AJ (2012). Impact of preoperative ureteral stenting on stone-free rates of ureteroscopy for nephroureterolithiasis: a matched-paired analysis of 286 patients. Urology..

[32] Allard WJ, Matera J, Miller MC, Repollet M, Connelly MC, Rao C (2004). Tumor cells circulate in the peripheral blood of all major carcinomas but not in healthy subjects or patients with nonmalignant diseases. Clin Cancer Res.

[33] Balic M, Dandachi N, Hofmann G, Samonigg H, Loibner H, Obwaller A (2005). Comparison of two methods for enumerating circulating tumor cells in carcinoma patients. Cytometry B Clin Cytom.

[34] Deng Z, Wu Y, Ma W, Zhang S, Zhang YQ (2015). Adoptive T-cell therapy of prostate cancer targeting the cancer stem cell antigen EpCAM. BMC Immunol.

[35] Groth A, Salnikov AV, Ottinger S, Gladkich J, Liu L, Kallifatidis G (2012). New gene-immunotherapy combining TRAIL-lymphocytes and EpCAMxCD3 Bispecific antibody for tumor targeting. Clin Cancer Res.

[36] Vallera DA, Zhang B, Gleason MK, Oh S, Weiner LM, Kaufman DS (2013). Heterodimeric bispecific single-chain variable-fragment antibodies against EpCAM and CD16 induce effective antibody-dependent cellular cytotoxicity against human carcinoma cells. Cancer Biother Radiopharm.

[37] Kallakury BV, Yang F, Figge J, Smith KE, Kausik SJ, Tacy NJ (1996). Decreased levels of CD44 protein and mRNA in prostate carcinoma. Correlation with tumor grade and ploidy. Cancer..

[38] Nagabhushan M, Pretlow TG, Guo YJ, Amini SB, Pretlow TP, Sy MS (1996). Altered expression of CD44 in human prostate cancer during progression. Am J Clin Pathol.

[39] Griebling T, Palechek P, Cohen M (1997). Immunohistochemical and soluble expression of CD44 in primary and metastatic human prostate cancers. Int J Oncol.

[40] De Marzo AM, Bradshaw C, Sauvageot J, Epstein JI, Miller GJ (1998). CD44 and CD44v6 downregulation in clinical prostatic carcinoma: relation to Gleason grade and cytoarchitecture. Prostate..

[41] Noordzij MA, van Steenbrugge GJ, Schroder FH, Van der Kwast TH (1999). Decreased expression of CD44 in metastatic prostate cancer. Int J Cancer.

[42] Aaltomaa S, Lipponen P, Ala-Opas M, Kosma VM (2001). Expression and prognostic value of CD44 standard and variant v3 and v6 isoforms in prostate cancer. Eur Urol.

[43] Ekici S, Ayhan A, Kendi S, Ozen H (2002). Determination of prognosis in patients with prostate cancer treated with radical prostatectomy: prognostic value of CD44v6 score. J Urol.

[44] Tei H, Miyake H, Harada K, Fujisawa M (2014). Expression profile of CD44s, CD44v6, and CD44v10 in localized prostate cancer: effect on prognostic outcomes following radical prostatectomy. Urol Oncol.

[45] Moura CM, Pontes J, Reis ST, Viana NI, Morais DR, Dip N (2015). Expression profile of standard and variants forms of CD44 related to prostate cancer behavior. Int J Biol Markers.

[46] Patrawala L, Calhoun T, Schneider-Broussard R, Li H, Bhatia B, Tang S (2006). Highly purified CD44+ prostate cancer cells from xenograft human tumors are enriched in tumorigenic and metastatic progenitor cells. Oncogene..

[47] Patrawala L, Calhoun-Davis T, Schneider-Broussard R, Tang DG (2007). Hierarchical organization of prostate cancer cells in xenograft tumors: the CD44+alpha2beta1+ cell population is enriched in tumor-initiating cells. Cancer Res.

[48] Hurt EM, Kawasaki BT, Klarmann GJ, Thomas SB, Farrar WL (2008). CD44+ CD24(−) prostate cells are early cancer progenitor/stem cells that provide a model for patients with poor prognosis. Br J Cancer.

[49] Huang WY, Lin JN, Hsieh JT, Chou SC, Lai CH, Yun EJ (2016). Nanoparticle targeting CD44-positive cancer cells for site-specific drug delivery in prostate cancer therapy. ACS Appl Mater Interfaces.

[50] Mahira S, Kommineni N, Husain GM, Khan W (2019). Cabazitaxel and silibinin co-encapsulated cationic liposomes for CD44 targeted delivery: a new insight into nanomedicine based combinational chemotherapy for prostate cancer. Biomed Pharmacother.

[51] Wei J, Sun J, Liu Y (2019). Enhanced targeting of prostate cancer-initiating cells by salinomycin-encapsulated lipid-PLGA nanoparticles linked with CD44 antibodies. Oncol Lett.

[52] Miller AM, Lundberg K, Ozenci V, Banham AH, Hellstrom M, Egevad L (2006). CD4+CD25high T cells are enriched in the tumor and peripheral blood of prostate cancer patients. J Immunol.

[53] Sfanos KS, Bruno TC, Maris CH, Xu L, Thoburn CJ, DeMarzo AM (2008). Phenotypic analysis of prostate-infiltrating lymphocytes reveals TH17 and Treg skewing. Clin Cancer Res.

[54] Lopez-Bujanda Z, Drake CG (2017). Myeloid-derived cells in prostate cancer progression: phenotype and prospective therapies. J Leukoc Biol.

[55] Pasero C, Gravis G, Guerin M, Granjeaud S, Thomassin-Piana J, Rocchi P (2016). Inherent and tumor-driven immune tolerance in the prostate microenvironment impairs natural killer cell antitumor activity. Cancer Res.

[56] Gannon PO, Poisson AO, Delvoye N, Lapointe R, Mes-Masson AM, Saad F (2009). Characterization of the intra-prostatic immune cell infiltration in androgen-deprived prostate cancer patients. J Immunol Methods.

[57] Kalina JL, Neilson DS, Comber AP, Rauw JM, Alexander AS, Vergidis J (2017). Immune modulation by androgen deprivation and radiation therapy: implications for prostate cancer immunotherapy. Cancers (Basel).

