# Supplementary material for: Targeting prostate cancer stem-like cells by an immunotherapeutic platform based on immunogenic peptide-sensitized dendritic cells-cytokine-induced killer cells
Source: Stem Cell Res Ther. 2020 Mar 17;11:123. doi: 10.1186/s13287-020-01634-6 (PMC7079411; doi:10.1186/s13287-020-01634-6)
Supplement: Supplementary file 1 — Additional file 1: Tables S1 and S2. Sequence information of CD44- and EpCAM-derived synthetic peptides and nucleotide sequence of PCR primers used. [file 13287_2020_1634_MOESM1_ESM.docx]

| **Name** | **Location** | **Amino acid sequence** | **Number of residues** |
| --- | --- | --- | --- |
| CD44-P1 | 28-39 | AGVFHVEKNGRY | 12 |
| CD44-P2 | 722-732 | DQFMTADETRN | 11 |
| CD44-P3 | 196-207 | YTFSTVHPIPDE | 12 |
| EpCAM-P1 | 15-23 | AATATFAAA | 9 |
| EpCAM-P2 | 155-166 | KPYDSKSLRTAL | 12 |
| EpCAM-P3 | 206-214 | DVDIADVAY | 9 |

**Supplementary Tables S1 and S2. Sequence information of CD44- and EpCAM-derived synthetic peptides and nucleotide sequence of PCR primers used**

| **Gene name** | **Forward (5′-3′)** | **Reverse (5′-3′)** |
| --- | --- | --- |
| *CD44* | GCGGCTCCTCCAGTGAAA | AGCCTGCTGAGATGGTATTT |
| *EPCAM* | GCAGGGTCTAAAAGCTGGTG | CCCTATGCATCTCACCCATC |
|  |  |  |
| *ACTB (β-actin)* | ATGGATGATGATATCGCCGCG | CTCCATGTCGTCCCAGTTGGT |
